# MET-targeted therapies for the treatment of non-small-cell lung cancer: A systematic review and meta-analysis

**DOI:** 10.3389/fonc.2022.1013299

**Published:** 2022-10-27

**Authors:** Linrui Xu, Faping Wang, Fengming Luo

**Affiliations:** ^1^ Department of Pulmonary and Critical Care Medicine, West China Hospital, Sichuan University, Chengdu, Sichuan, China; ^2^ Laboratory of Pulmonary Immunology and Inflammation, Frontiers Science Center for Disease-related Molecular Network, West China Hospital, Sichuan University, Chengdu, Sichuan, China; ^3^ Clinical Research Center for Respiratory Disease, West China Hospital, Sichuan University, Chengdu, Sichuan, China

**Keywords:** non-small cell lung cancer, MET inhibitors, activity, safety, meta-analysis

## Abstract

**Background:**

Dysregulation of the mesenchymal epithelial transition (MET) pathway contributes to poor clinical outcomes in patients with non-small cell lung cancer (NSCLC). Numerous clinical trials are currently investigating several therapies based on modulation of the MET pathway.

**Objectives:**

This study aimed to systematically evaluate the activity and safety of MET inhibitors in patients with NSCLC.

**Methods:**

We searched PubMed, Embase, and the Cochrane Library from inception to June 02, 2022. The objective response rate (ORR) and disease control rate (DCR) were extracted as the main outcomes and pooled using the weighted mean proportion with fixed- or random-effects models in cases of significant heterogeneity (*I* ^2^>50%). Safety analysis was performed based on adverse events reported in all studies.

**Results:**

Eleven studies (882 patients) were included in the meta-analysis. The pooled ORR was 28.1% (95% confidence interval [CI], 0.223–0.354), while the pooled DCR was 69.1% (95% CI, 0.631–0.756). ORRs were higher for tepotinib (44.7% [95% CI, 0.365–0.530]) and savolitinib (42.9% [95% CI, 0.311–0.553]) than for other types of MET inhibitors. Patients with NSCLC with exon 14 skipping exhibited higher ORRs (39.3% (95% CI, 0.296–0.522)) and DCRs (77.8% (95% CI, 0.714–0.847)) than those with MET protein overexpression or amplification. Intracranial response rate and intracranial disease control rates were 40.1% (95% CI, 0.289–0.556) and 95.4% (95% CI, 0.892–0.100), respectively. Adverse events were mild (grade 1 to 2) in 87.2% of patients. Common adverse events above grade 3 included lower extremity edema (3.5% [95% CI, 0.027–0.044]), alanine aminotransferase (ALT) elevation (2.4% [95% CI, 0.014–0.033]), and lipase elevation (2.2% [95% CI, 0.016–0.031]).

**Conclusion:**

MET inhibitors, which exhibited a satisfactory safety profile in the current study, may become a new standard of care for addressing MET dysregulation in patients with advanced or metastatic NSCLC, and even in those with brain metastases, particularly tepotinib, savolitinib and capmatinib. Further randomized trials are required to establish standard predictive biomarkers for MET therapies and to compare the effects of different MET inhibitors in NSCLC with MET dysregulation.

## 1 Introduction

Lung cancer is the leading cause of cancer-related death worldwide, resulting in an estimated 1.6 million deaths each year (1, 2). Non-small-cell lung cancer (NSCLC) is a predominant lung cancer subtype that accounts for nearly 85% of lung cancer cases, with an annual global incidence that continues to increase (3, 4). The five-year overall survival rate for NSCLC is poor, decreasing from 68% in patients with stage IB disease to 0–10% in those with stage IVA–IVB disease (5). Understanding the pathophysiology of NSCLC and detecting relevant mutations is crucial for developing effective therapeutic strategies. However, NSCLC is molecularly heterogeneous (6), and its development and progression have been associated with various oncogenic drivers (7–9). Current diagnostic standards for NSCLC are based on the detection of epidermal growth factor receptor (EGFR), v-raf murine sarcoma viral oncogene homolog B1 (BRAF), and mesenchymal epithelial transition (MET) mutations, as well as analyses of anaplastic lymphoma kinase (ALK), ROS proto-oncogene receptor tyrosine kinase 1 (ROS1), and neurotrophic receptor tyrosine kinase (NTRK) translocations. MET dysregulation is notable in that extreme increases in MET activity can induce tumorigenesis and lead to invasion, proliferation, angiogenesis, metastatic spread of tumors, and resistance to cancer treatments (10, 11).

Dysregulation of the MET pathway in NSCLC is thought to occur *via* various mechanisms, including protein overexpression, gene mutation, amplification, and rearrangement (12). The reported prevalence of MET amplification in patients with NSCLC ranges from 1–5% (13–15), while that of MET protein overexpression ranges from 25–75% (16–18). In patients with non-squamous NSCLC, the rate of MET mutations ranges from 2–4% (15, 19, 20), although such mutations are clearly overrepresented among older adults, in whom the prevalence is comparable to that observed in patients with ALK-rearranged lung cancer (21). Studies have demonstrated that both MET overexpression and amplification are related to poor prognosis in patients with NSCLC (22–25), and MET amplification appears to be an independent marker of poor outcome following surgically resection of NSCLC (26–28). In a series of 687 Asian patients with resected NSCLC, MET alterations were poor prognostic factors for overall survival (OS) (27). A review by Pilotto et al. highlighted MET exon 14 skipping alterations as potential oncogenic targets in lung cancer given their ability to drive the activity of MET inhibitors in molecularly selected patients (29). Based on this evidence, the MET pathway has been explored as a potential therapeutic target NSCLC drug development. Within the last decade, several MET inhibitors have been developed and are undergoing investigation in clinical trials (30–34), including tyrosine kinase inhibitors (TKIs), monoclonal antibodies (mAb), and antibody-drug conjugates (ADCs). TKIs that target the MET pathway are generally divided into two types (I and II). Type I inhibitors are adenosine triphosphate (ATP) competitors that bind to the ATP-binding pocket of the active form (DFG-in), whereas type II TKIs are ATP competitors that bind to the inactive state (DFG-out), resulting in a configuration that may benefit those with acquired resistance to type I TKIs (35). Some of the prototypical drugs include crizotinib (type I), capmatinib (type I), tepotinib (type I), savolitinib (type I), cabozantinib (type II), glesatinib (type II), and merestinib (type II). Monoclonal antibodies inhibiting the MET pathway target the extracellular domain, leading to signaling inhibition (36), and include drugs such as onartuzumab and emibetuzumab. Mechanistically, ADCs such as telisotuzumab vedotin and amivantamab exert effects following antibody binding *via* more targeted, direct delivery of a cytotoxic payload to the tumor cells, limiting any resistance mechanisms that may be related to intracellular signaling, such as MET amplification in EGFR TKI resistance (37).

A 2015 study reported dramatic and durable partial responses (PRs) to crizotinib in patients with NSCLC harboring MET alterations (38). Durable PRs to capmatinib have also been reported in patients with advanced NSCLC exhibiting MET dysregulation (15). Subsequent case reports have confirmed these findings using different MET inhibitors across NSCLC histologies (23, 39–41). Given the promising responses in patients treated with MET inhibitors in some clinical trials, the Japanese Ministry of Health, US FDA, and China National Medical Products Administration approved tepotinib, capmatinib, and savolitinib for the treatment of NSCLC with MET dysregulation in 2020. This decision not has not only bridged the gap in the treatment landscape for NSCLS with MET dysregulation but has also ushered in a new era for MET inhibitors. However, despite the promise of MET inhibitors for NSCLC with MET dysregulation in most clinical trials, several studies have reported low treatment response rates, and the safety and precise mechanisms underlying the effects of MET inhibitors in the treatment of NSCLC remain unclear. Therefore, to promote optimal clinical treatment, we conducted a meta-analysis of studies related to the activity and safety of MET inhibitors in patients with NSCLC.

## 2 Methods

This study was registered at PROSPERO under registration number CRD42022341285 and aligned with the Preferred Reporting Items for Systematic Reviews and Meta-Analyses (PRISMA) guidelines during all stages of design, implementation, and reporting (42).

### 2.1 Literature searching

An exhaustive literature search involving computer-assisted and manual methods was conducted. Two independent investigators (LX and FW) conducted a systematic literature search of PubMed, Embase and the Cochrane library using the key words “c-Met inhibitor” and “non-small-cell lung cancer”. The last date of the search was June 02, 2022. The detailed search strategy is presented in **Supplementary Table S1**.

### 2.2 Eligibility criteria

Studies with relevant information on patient characteristics, treatment interventions, and outcomes were included. Eligibility was limited to articles reporting the result of clinical trials and published in English. The inclusion criteria were as follows: 1) histologically confirmed diagnosis of locally advanced or metastatic NSCLC with dysregulation of the MET pathway; 2) single-agent treatment with a MET inhibitor; 3) assessments of objective response rate (ORR, defined as the proportion of patients with a complete or partial response) and/or disease control rate (DCR, defined as the proportion of patients with a complete response, a partial response, or stable disease) based on the guidelines provided by Response Evaluation Criteria in Solid Tumors (RECIST) version 1.1 (43); and 4) adverse events recorded and graded by the investigators according to the National Cancer Institute Common Terminology Criteria for Adverse Events (AEs). All reported adverse events associated with drug treatment were included in the safety assessment. Articles for which only abstracts could be located were excluded.

### 2.3 Study selection and data extraction

Two reviewers independently performed the search, study selection, and data extraction steps. In case of discrepancies, consensus was reached *via* discussion. Two reviewers independently screened titles and abstracts from the data sources based on the eligibility criteria mentioned above. The full texts of the potentially relevant articles were then reviewed thoroughly to guarantee eligibility criteria. We recorded the following information from the original literature: (1) first author; (2) year of publication; (3) study design; (4) baseline patient data, including the number of patients who met the inclusion criteria, type of MET dysregulation, sex, and age; (5) type, dose, and schedule of MET inhibitor treatment; (6) Eastern Cooperative Oncology Group performance status (ECOG PS); (7) response/outcome; and (8) AEs.

### 2.4 Quality assessment

We used the Methodological Index for Non-randomized Studies (MINORS) (44) to assess the quality of non-randomized studies.

### 2.5 Statistical synthesis

Baseline patient characteristics, treatment responses, and AEs were analyzed for all enrolled studies. The ORR and DCR of NSCLC with MET-dysregulation were expressed as mean rates with 95% confidence intervals (95% CI). A subgroup analysis was performed based on different types of MET inhibitors, different types of MET dysregulation, and therapy types (monotherapy or combination therapy). The chi-square test (*Q* statistic) and *I (* 2 *)* statistics were used to assess heterogeneity (45). If *P* ≥ 0.10 and/or *I (* 2 *)* ≤ 50%, heterogeneity was considered low, and a fixed-effects model was selected. In other cases, random-effects models were used. Sensitivity analysis was performed by individually excluding each study with high heterogeneity from the pooled results. The risk of publication bias was determined using funnel plots and Egger’s test (46). Statistical significance was set at *P < 0.05*. AEs were extracted from all studies for the pooled safety analysis. We used logit transformation to perform a meta-analysis of raw proportions with a continuity correction of 0.5 in studies with zero cell frequencies. All analyses were performed using R programming language (package meta, version 3.6.1).

## 3 Results

### 3.1 Search results and characteristics of included studies

**Figure 1:**
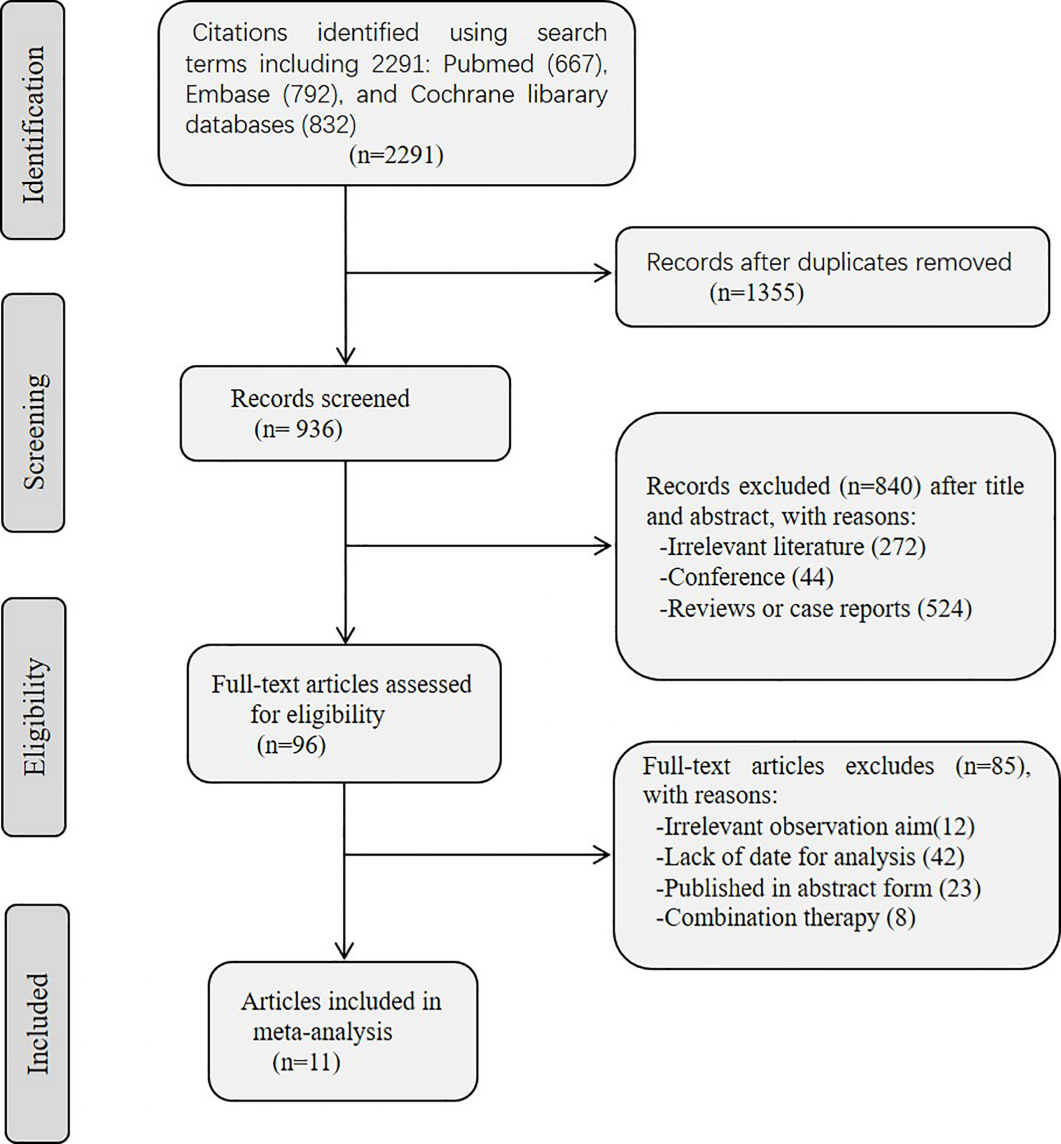
Flow diagram of study selection process for this systematic review and meta-analysis.

A flow chart of the screening process is shown in **Figure 1**. A total of 2,291 articles were identified *via* the database search. Among the selected studies, 1,355 duplicated articles were excluded using Endnote software. After exclusion based on titles and abstracts, 96 full-text articles were reviewed. After a full-text screening, 11 publications (882 patients) were included in this systematic review. The classifications and features of the included studies are presented in **Table 1**. A total of 3 (31, 34, 47) studies were phase I trials, while 8 (48–55) studies were phase II trials. All patients were diagnosed with advanced or metastatic NSCLC. Types of MET dysregulation included MET amplification, exon 14 skipping, and MET protein overexpression. One study included patients with MET amplification (53); three included patients with exon 14 skipping (34, 54, 55), four included patients with MET amplification and exon 14 skipping (49–52), one include patients with MET amplification and MET protein overexpression (31), and two included patients with all three types of MET dysregulation (47, 48). Patients were treated with capmatinib in three studies (48–50), crizotinib in four studies (34, 51–53), SAR125844 in one study (31), savolitinib in one studies (54), telisotuzumab vedotina in one studies (47), and tepotinib in one studies (55). Overall, most Eastern Cooperative Oncology Group performance status (ECOG PS) scores were 0 or 1. The characteristics of the included studies are described in detail in **Table 1**.

**Table 1:** Baseline clinical characteristics of included studies.

Study	Study design	Ethnicity	Enrolled Patient (n)	Sex(F, %)	Mean age(yrs)	Cancer type	MET dysregulation			MET inhibitor therapy	ECOG PS		
							Amplification (%)	Exon 14 skipping (%)	MET protein overexpression		0	1	2
Schuler (2020)	Phase II trial	Caucasian and Asian	55	40.0%	60 (29-84)	Stage IIIB or IV NSCLC	80.0%	7.3%	98.2%	Capmatinib 400 mg (tablets) or 600 mg (capsules) twice daily	35.0%	62.0%	3.0%
Wolf (2020)	Phase II trial	NA	364	36.4%	NA	Stage IIIB or IV NSCLC	58.5%	41.5%	–	Capmatinib 400 mg twice daily	26.4%	73.6%	0%
Dagogo-Jack (2021)	Phase II trial	Mainly White	20	60.0%	70 (57-88)	Stage IIIB to V NSCLC	25.0%	75.0%	–	Capmatinib 400 mg twice daily	NA	NA	NA
Landi (2019)	Phase II trial	NA	26	35.0%	56 (39–78)	Advanced NSCLC	61.5%	38.5%	–	Crizotinib 250 mg twice daily	42.0%	50.0%	8.0%
Moro-Sibilot (2019)	Phase II trial	NA	53	NA	NA	Advanced/metastatic NSCLC	47.2%	52.8%	–	Crizotinib 250 mg twice daily	18.9%	50.9%	30.2%
Drilon (2020)	Phase I trial	Mainly White and Asian	69	58.0%	72(34-91)	Advanced NSCLC	–	100%	–	Crizotinib 250 mg twice daily	28.0%	71.0%	1.0%
Camidge (2021)	Phase II trial	Mainly White	38	44.7%	66.5(42-88)	Advanced NSCLC	100%	–	–	Crizotinib 250 mg twice daily	28.9%	57.9%	13.2%
Angevin (2017)	Phase I trial	NA	29	55.2%	62.0 (43-77)	Advanced NSCLC	75.9%	–	24.1%	Once-weekly SAR125844 570 mg/m2	10.3%	89.7%	0%
Lu (2021)	Phase II trial	Asian	70	41.0%	68·7 (65·4–74·7)	Advanced/metastatic NSCLC	–	100%	–	Savolitinib 600 mg (bodyweight ≥50 kg) or 400 mg (bodyweight <50 kg) of once daily	17.0%	82.0%	1.0%
Camidge (2021)	Phase I trial	NA	40	40.0%	66 (40–86)	Advanced NSCLC	5.0% (Concurrent overexpression)	3.0% (Concurrent overexpression)	100%	Telisotuzumab vedotina≥1.6 mg/kg once every 2 weeks or ≥2.4 mg/kg once every 3 weeks	18.0%	82.0%	0.0%
Le (2022)	Phase II trial	White and Asian	152	52.0%	73.1 (41–94)	Advanced/metastatic NSCLC	–	100%	–	Tepotinib 500 mg once daily	27.0%	73.0%	0%

MET, mesenchymal epithelial transition; NSCLC, non-small cell lung cancer. NA, not available.

### 3.2 Quality assessment

All non-randomized studies assessed using the MINORS index had scores ranging from 13 to 15 points, which were considered acceptable for the present meta-analysis (**Table 2**).

**Table 2:** Quality assessment of included studies.

MINORS index for included non-randomized studies.									
Study	I	II	III	IV	V	VI	VII	VIII	Total
Angevin (2017)	2	1	2	2	2	2	2	1	14
Landi (2019)	2	1	2	2	2	2	2	1	14
Moro-Sibilot (2019)	2	1	2	2	2	2	2	1	14
Schuler (2020)	2	2	2	2	2	2	2	1	15
Wolf (2020)	2	1	2	2	2	2	1	1	13
Drilon (2020)	2	2	2	2	2	2	2	1	15
Dagogo-Jack (2021)	2	1	2	2	2	2	2	1	14
Camidge (2021)	2	2	2	2	2	2	2	1	15
Lu (2021)	2	2	2	2	2	2	2	1	15
Camidge (2021)	2	2	2	2	2	2	2	1	15
Le (2022)	2	1	2	2	2	2	1	1	13

numbers I-VIII in heading signified: I, a clearly stated aim; II, inclusion of consecutive patients; III, prospective collection of data; IV, endpoints appropriate to the aim of the study; V, unbiased assessment of the study endpoint; VI, follow-up period appropriate to the aim of the study; VII, loss of follow up less than 5%; VIII, prospective calculation of the study size.

### 3.3 Response rate

#### 3.3.1 Objective response rate

**Figure 2:**
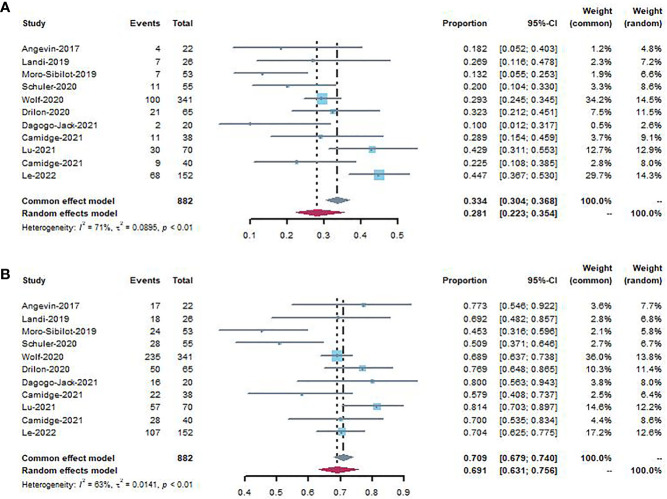
Forest plot showing ORR **(A)** and DCR **(B)** to patients using MET inhibitors.

The pooled ORR was evaluated in 11 studies involving 882 patients with NSCLC. When all studies were considered, objective responses were reported in 270 patients (270/882, 30.6%) after MET inhibitor treatment. The ORR ranged from 10.0% to 44.7%, and the random-effects model was selected given the heterogeneity among the studies (*I* (2 *)* = 71%, *P* < 0.01). The pooled rate was 28.1% (95% CI, 0.223–0.354) (**Figure 2A**).

**Figure 3:**
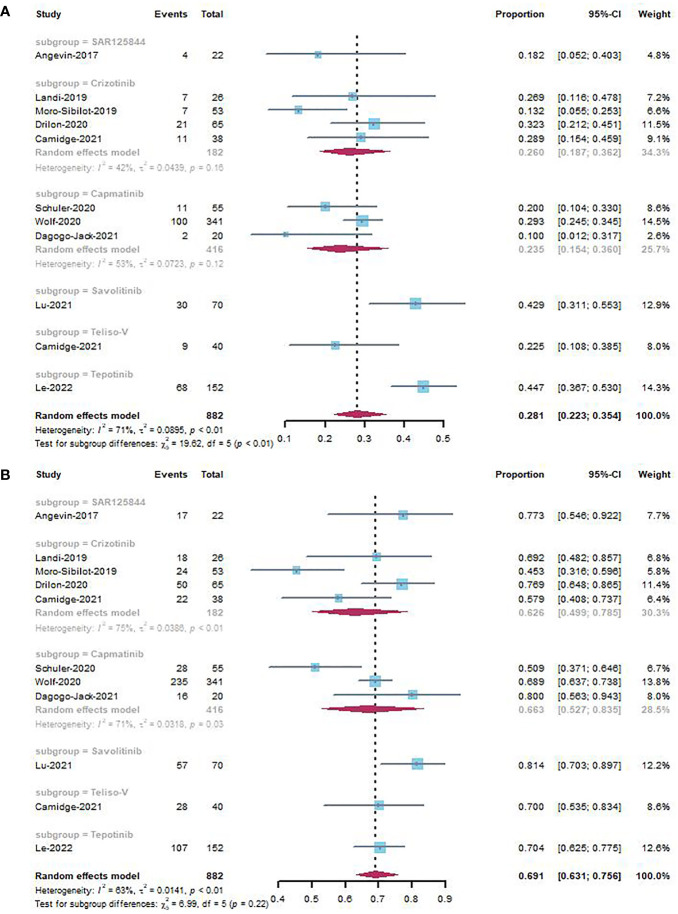
Subgroup analyses by different MET inhibitors of ORR **(A)** and DCR **(B)** in NSCLC studies.

The forest plots for the subgroup analyses of studies involving different MET inhibitors including SAR125844, crizotinib, capmatinib, savolitinib, telisotuzumab vedotin, and tepotinib are shown in **Figure 3A**. SAR125844, crizotinib, capmatinib, savolitinib, telisotuzumab vedotin, and tepotinib were associated with ORRs of 18.2% (95% CI, 0.052–0.403), 26.0% (95% CI, 0.187–0.362), 23.5% (95% CI, 0.154–0.360),42.9% (95% CI, 0.311–0.553), 22.5% (95% CI, 0.108–0.385), and 44.7% (95% CI, 0.365–0.530) without heterogeneity, respectively.

**Figure 4:**
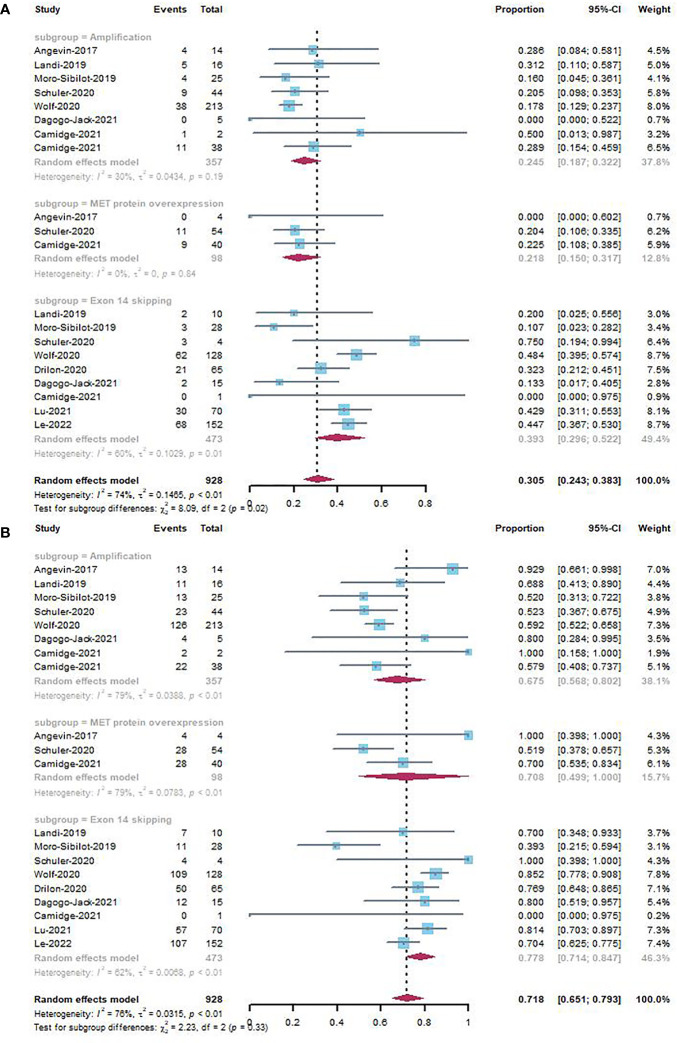
Subgroup analyses by different MET dysregulation of ORR **(A)** and DCR **(B)** in NSCLC studies.

The forest plots for subgroup analyses of studies involving different types of MET dysregulation, including exon 14 skipping, MET protein overexpression, and amplification, are shown in **Figure 4A**. Among patients with NSCLC, MET amplification and MET protein overexpression were associated with ORRs of 24.5% (95% CI, 0.187–0.322) and 21.8% (95% CI, 0.150–0.317) without heterogeneity, respectively. In patients with exon 14 skipping, the ORR was 39.3% (95% CI, 0.296–0.522, *I^2 =^* 60%).

#### 3.3.2 Disease control rate

All studies were included in the analysis of DCR (**Figure 2B**), which ranged from 45.3% to 81.4%. The pooled DCR was 69.1% (95% CI, 0.631–0.756), with high heterogeneity among studies (*I* (2 *)* = 63%, *P* < 0.01).

Forest plots for subgroup analyses are shown in **Figure 3B**. SAR125844, crizotinib, capmatinib, savolitinib, telisotuzumab vedotin, and tepotinib were associated with DCRs of 77.3% (95% CI, 0.546–0.922), 62.6% (95% CI, 0.499–0.785), 66.3% (95% CI, 0.527–0.835), 81.4% (95% CI, 0.703–0.897), 70.0% (95% CI, 0.535–0.834), and 70.4% (95% CI, 0.625–0.775), respectively.

The forest plots for subgroup analyses of studies involving different types of MET dysregulation, including exon 14 skipping, MET protein overexpression, and amplification, are shown in **Figure 4B**. The DCRs for MET amplification, MET protein overexpression, and exon 14 skipping were 67.5% (95% CI, 0.568–0.802, *I^2 =^* 75%), 70.8% (95% CI, 0.499–1.000, *I^2 =^* 79%), and 77.8% (95% CI, 0.714–0.847, *I^2 =^* 62%), respectively, with high heterogeneity among studies.

#### 3.3.3 Central nervous system (CNS) activity

**Figure 5:**
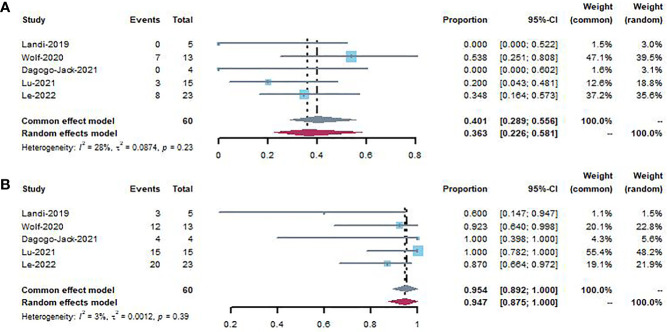
Forest plot showing intracranial response **(A)** and intracranial disease control **(B)** to patients using MET inhibitors.

**Figure 5** shows the forest plot for intracranial response and intracranial disease control rates. The pooled intracranial response rate and intracranial disease control rate were 40.1% (95% CI, 0.289–0.556; *I^2 =^* 28%) and 95.4% (95% CI, 0.892–0.100; *I^2 =^* 3%) without heterogeneity, respectively.

### 3.4 Adverse Events

All patients treated with MET inhibitors were evaluated for AEs, some of which were not included in the pooled analysis of response outcomes because the treatment response data were unavailable. There were 10 (31, 34, 47–51, 53–55) studies (829 patients) reporting 2,103 AEs associated with MET inhibitor treatment, most of which were mild. Five studies (34, 49, 50, 54, 55) reported 7 deaths due to MET inhibitor treatment. One study (52) did not provide relevant information on AEs. We evaluated AEs related to MET inhibitor treatment according to the National Cancer Institute Common Terminology Criteria for Adverse Events, which are shown in **Table 3**. A total of 829 patients from ten studies were included in the safety evaluation. In these patients, 87.2% AEs were mild to moderate (grade 1–2), the most common of which included lower extremity edema, nausea, fatigue, vomiting, diarrhea, anorexia, alanine aminotransferase (ALT) elevation, and creatinine elevation. A total of 270 grade 3 or 4 AEs were observed, the most common of which included lower extremity edema, ALT elevation, lipase elevation, fatigue, AST elevation, amylase elevation, vomiting, and nausea. Seven deaths were associated with MET inhibitor treatment in 829 patients: three due to pneumonitis, two in patients with interstitial lung disease, one in a patient with dyspnea, and one in a patient with tumor lysis syndrome. The pooled rates of ≥grade 3 common AEs were 3.5% (95% CI, 0.027-0.044) for lower extremity edema, 2.4% (95% CI, 0.014–0.033) for ALT elevation, and 2.2% (95% CI, 0.016–0.031) for lipase elevation (**Table 4**).

**Table 3:** Adverse events observed after MET inhibitors in NSCLC patients.

Study	Evaluation Patients, n	Adverse events, n	Adverse events, (n)				
			Grade 1	Grade 2	Grade 3	Grade 4	Grade 5
Dagogo-Jack (2021)^*^	20	52	33	10	7	0	2
Schuler (2020)^*^	55	88	80 (including Grade 2)	–	8 (including Grade 4)	–	0
Wolf (2020)^*^	364	708	589 (including Grade 2)	–	118 (including Grade 4)	–	1
Camidge (2021)^*^	37	54	42 (including Grade 2)	–	12	0	0
Camidge (2021)^*^	52	59	46 (including Grade 2)	–	13 (including Grade 4)	–	0
Drilon (2020)^*^	69	225	168	50	6	0	1
Landi (2019)^*^	26	75	68 (including Grade 2)	–	7 (including Grade 4)	–	0
Lu (2021)^*^	70	199	172 (including Grade 2)	–	26 (including Grade 4)	–	1
Moro-Sibilot (2019)^na^	53	NA	–	–	–	–	–
Angevin (2017)^*^	29	101	95 (including Grade 2)	–	6 (including Grade 4)	–	0
Le (2022)^*^	255	542	480	–	60	–	2

NA, not available.

*, Treatment-emergent adverse event.

Grade1-2: Mild to moderate;

Grade 3: Severe but not immediately life-threatening;

Grade 4: Life-threatening consequence;

Grade 5: Death.

**Table 4:** Pooled results of common AEs of any grade and ≥grade 3.

AEs	Any grade		AEs	≥grade 3	
	Proportion % (95 % CI)	*I^2^*, %		Proportion % (95 % CI)	*I^2^*, %
Lower-extremity edema	17.1 (12.8-22.8)	80	Lower-extremity edema	3.5 (2.7-4.4)	18
Nausea	14.0 (10.7-18.2)	77	ALT elevation	2.4 (1.4-3.3)	16
Fatigue	10.2 (6.9-15.5)	85	Lipase elevation	2.2 (1.6-3.1)	43
Vomiting	7.6 (5.0-11.6)	79	Fatigue	1.6 (0.8-3.9)	61
Diarrhea	7.1 (5.1-9.9)	65	AST elevation	1.3 (0.6-2.6)	53
Anorexia	6.2 (5.2-7.4)	45	Amylase elevation	1.3 (0.9-2.1)	4
ALT elevation	5.3 (3.4-8.4)	77	Vomiting	1.0 (0.6-1.6)	0
Creatinine elevation	2.8 (1.2-6.6)	69	Nausea	1.0 (0.6-1.6)	0

AE, adverse events; ALT, Alanine transaminase; AST, Aspartate aminotransferase.

### 3.5 Sensitivity analysis

**Figure 6:**
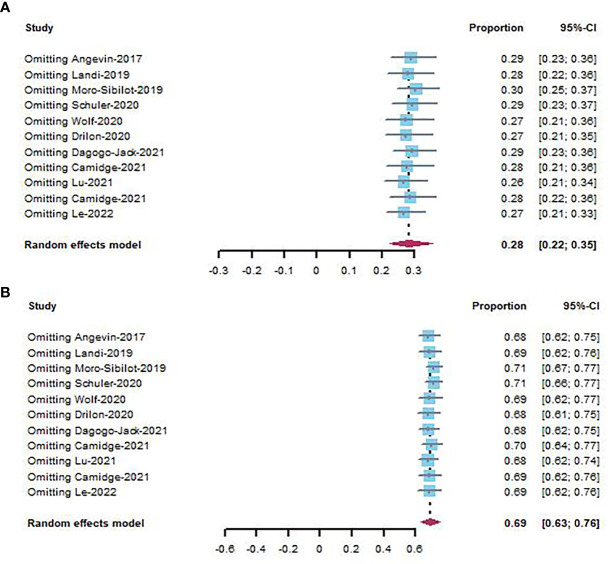
Sensitivity analysis of ORR **(A)** and DCR **(B)** in NSCLC studies.

Sensitivity analysis was performed by removing individual studies one by one from the pooled results with high heterogeneity. The pooled analysis of ORR and DCR did not change significantly when studies were omitted, indicating that our combined results are reliable (**Figure 6**).

### 3.6 Publication bias

**Figure 7:**
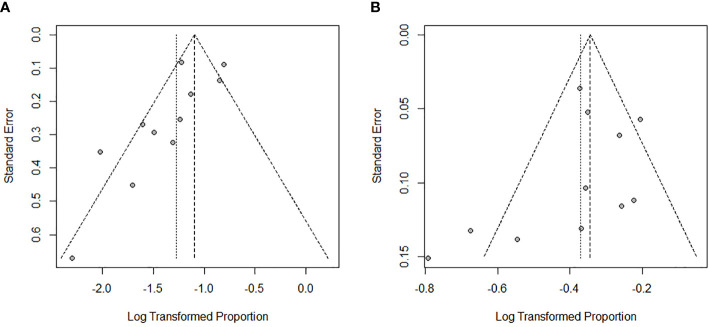
Egger funnel plot of ORR **(A)** and DCR **(B)**.

We used the Egger’s test and funnel plots to evaluate the publication bias in studies included. The results of the Egger’s test showed no evidence of publication bias in the studies on ORR (*P* =0.13) (**Figure 7A**) and DCR (*P* =0.29) (**Figure 7B**). This was consistent with the shape of funnel plots which had a good symmetry.

## 4 Discussion

In this meta-analysis, we examined the activity and safety of MET inhibitors in patients with NSCLC exhibiting MET dysregulation based on the results of clinical trials. In our study including 882 patients with NSCLC, the estimated ORR was 28.1%, while the DCR was 69.1%. These results highlight the promise of MET inhibitors in patients with advanced/metastatic NSCLC with MET dysregulation, especially tepotinib and savolitinib. Moreover, patients with exon 14 skipping responded best to MET inhibitors, with an ORR of 39.3% and a DCR of 77.8%. Among 829 cases, 2,103 AEs associated with MET-inhibitor-treatment were reported, 87.2% of which were mild (grade 1–2), indicating that MET inhibitors are tolerated by most patients. The most common adverse events above grade 3 included lower extremity edema (3.5%), ALT elevation (2.4%), and lipase elevation (2.2%).

Although the overall ORR was not very high in patients treated with MET inhibitors, satisfactory overall DCRs were observed. In general, therapeutic responses vary widely for different MET inhibitors in different trials, from 10.0% to 44.7% for ORR and 45.3% to 81.4% for DCR. This may be explained by the different biological value of each individual MET alteration (MET overexpression, amplification, and MET exon 14 skipping) and the definitions/methods used to detect MET alterations in different trials. It remains highly debated whether patients with MET overexpression or amplification can benefit from MET inhibitors; however, MET exon 14 skipping is now an established biomarker, and patients with such alterations have been shown to benefit from MET-targeted therapies (56). MET inhibitors against MET exon 14 skipping offer hope to affected patients, as previous studies have demonstrated a low response to immune-oncology drugs in this population (57). Our pooled treatment response results for the MET dysregulation subgroup indicated that patients with exon 14 skipping had a higher ORR (39.3%) and DCR (77.8%) than those with MET protein overexpression or amplification, which is consistent with current views regarding the therapeutic tractability of different MET alterations. Immunohistochemistry (IHC) results for MET protein overexpression and fluorescence *in situ* hybridization (FISH) results for MET amplification are continuous variables, meaning that the selection of cut-off points is crucial. Only patients with a very high level of MET protein overexpression or amplification may benefit from these treatments, and patients with an MET status below a given threshold may exhibited diminished responses at the individual level (58). The results of initial trials evaluating MET inhibitor efficiency focused on unselected NSCLC have been negative (59), which may also be explained by wide variations in responses. MET amplification leads to overexpression or constitutive kinase activation *via* multiplication of the MET gene and synthesis of MET protein in excess; thus, the level of gene amplification may act as an oncogenic driver in patients with NSCLC (12). Previous studies involving patients with MET-amplified advanced NSCLC have reported greater efficacy in tumors with a high gene copy number than in those with a low gene copy number, and clinical trials of capmatinib have revealed that a gene copy number (GCN) ≥ 6 is associated with satisfactory anti-tumor activity (48, 49). Unlike MET amplification, MET overexpression can reflect both genomic and non-genomic processes; thus, the lack of correlation between protein expression and genomic alterations indicates that MET protein overexpression may not be a reliable patient selection criterion for MET-targeted therapies (60). The use of targeted therapies in this population has produced disappointing results in the context of advanced NSCLC (61, 62). Our pooled ORR was 23.5% in patients with MET overexpression, which is similar to the rate observed in patients with MET amplification, mainly because these patients had concurrent MET amplification and MET exon 14 skipping. Thus, MET overexpression alone may not be a reliable biomarker for predicting the activity of MET inhibitors. Next-generation sequencing (NGS) for MET exon 14 skipping mutations and/or amplification, FISH for MET amplification, and IHC for MET overexpression are widely used to detect MET dysregulation. Establishing standard predictive biomarkers for MET therapies remains an urgently requirement, as it is important for clinicians to distinguish between the various mechanisms of MET dysregulation to ensure appropriate testing and prompt treatment with optimal methods.

Current MET-signaling-targeted therapeutic strategies include inhibiting kinase activity, preventing phosphotransferase activity, and blocking MET signaling (58, 63, 64). This study evaluated the benefits of MET inhibitors, including anti-MET antibodies (SAR125844) (31), TKIs (crizotinib, savolitinib, tepotinib, capmatinib) (34, 48–55), and ADC (telisotuzumab vedotin) (47). Notably, the efficiency of tepotinib and savolitinib in clinical trials has been promising, with ORRs reaching nearly 50%. Both drugs are selective c-MET TKIs that have been approved for the treatment of NSCLC with MET dysregulation in Japan and China. One possible reason for the positive results is the proper selection of patients. In the included trials, patients treated with savolitinib monotherapy (54) and tepotinib monotherapy (55) all had MET exon 14 skipping mutations, which appears to be the most promising subset with sensitivity to MET inhibitors. In a clinical trial of capmatinib monotherapy, Wolf et al. reported similar ORRs to these two drugs in patients with NSCLC harboring MET exon 14 skipping mutations, and the overall ORR in patients with both MET exon 14 skipping mutations and MET amplification was 29.3%. Capmatinib has been approved for the treatment of NSCLC with MET dysregulation in the US. The inclusion of patients with different types of MET dysregulation (mutations, amplification, and overexpression) may have influenced the overall treatment effect in previous studies. Capmatinib has exhibited satisfactory antitumor activity in patients with MET exon 14 skipping mutations or high-GCN MET amplification, particularly in those who had not received previous treatment (48, 49); Tepotinib and savolitinib have exhibited satisfactory clinical activity in patients with MET exon 14 skipping mutations, irrespective of previous systemic treatment, and in those with brain metastases (54, 55). Another study reported satisfactory antitumor activity following crizotinib treatment for NSCLC with high levels of MET amplification (MET-to-CEP7 ratio ≥4, ORR: 38.1%) and for NSCLC with exon 14 skipping mutations (53). In addition, SAR125844 has shown modest antitumor activity (ORR: 28.6%) in NSCLC with MET amplification (31). In addition to monotherapy, combination therapies have been investigated in several clinical trials. MET dysregulation is known to confer primary or secondary resistance against EGFR TKIs, and research has demonstrated that inhibiting MET expression through shRNA can restore sensitivity to EGFR-TKIs (65). Based on this rationale, patients with MET amplification or MET protein overexpression plus MET-driven EGFR TKI resistance (32, 33, 66) may benefit from a combination of savolitinib and tepotinib (savolitinib plus osimertinib, savolitinib plus gefitinib, and tepotinib plus gefitinib), even those with disease progression following prior treatment with an EGFR inhibitor. These results highlight the potential of savolitinib and tepotinib to become the new standard of care for NSCLC, especially in patients with MET exon 14 skipping mutations. The combination of MET TKIs and EGFR TKIs (osimertinib plus savolitinib, savolitinib plus gefitinib, tepotinib plus gefitinib) may also be promising for MET-driven EGFR TKI resistance. Other combination therapies have been associated with minimal activity (ORRs lower than 20%), including capmatinib plus gefitinib, capmatinib plus erlotinib, telisotuzumab vedotina plus nivolumab, onartuzumab plus erlotinib) (30, 47, 61, 67, 68). However, these results may have been affected by weak preclinical rationale or inadequate patient selection.

Brain metastases may occur in up to 20 to 40% of patients with stage IV NSCLC (69). In the pooled analysis of MET activity in the CNS within a small patient group (N = 60), an intracranial response was observed in 40.1% of patients, while intracranial disease control was observed in 95.4% of patients. Given the importance of CNS control in maintaining the best disease response and quality of life, confirmation of these preliminary findings in a larger population is crucial.

The current findings indicate that MET inhibitors are well-tolerated and safe in patients with NSCLC. AEs mainly included lower-extremity edema, nausea, fatigue, diarrhea, vomiting, anorexia, ALT elevation, and creatinine elevation, most of which were mild and persisted for a short time only. Peripheral edema and nausea are the most frequent AEs in TKI trials, while anemia and fatigue are the most frequent events in trials of mAb and ADC. In our analysis, only seven deaths were reported to be associated with MET-inhibitor treatment. Although antibodies have excellent target specificity and predictable pharmacological properties, their toxicity is similar to that of molecular inhibitors.

There was high heterogeneity (*I* (2 *)* = 71%, *P* < 0.01) among ORRs and DCRs (*I* (2 *)* = 63%, *P* < 0.01). Subgroup analyses of each drug indicated that ORRs were higher for savolitinib (ORR, 42.9%) and tepotinib (ORR, 44.7%) than for other MET inhibitors, while subgroup analyses based on MET dysregulation type indicated that both ORRs and DCRs were higher for patients with exon 14 skipping (ORR, 39.3%; DCR, 77.8%) than for those with MET protein overexpression or amplification. These findings support additional evidence regarding the heterogeneity of NSCLC.

This meta-analysis had several limitations. First, the number of patients included was small, and no RCTs were included in this review. The small sample size may have influenced the strength of our study. Second, all studies failed to compare the activity of different types of MET inhibitors, meaning that we were unable to provide unbiased head-to-head comparisons of treatment effects. Third, despite a careful electronic search of the literature databases, some publications may have been missed. Fourth, most of this evidence is based on phase I and II studies; therefore, phase III studies are warranted to confirm the activity and safety of MET inhibitors.

## 5 Conclusion

The results of the current systematic review and meta-analysis suggest that MET inhibitors, especially savolitinib and tepotinib, are promising treatment options for NSCLC. Based on our analysis, most patients exhibit good tolerance to MET inhibitors. However, considering the limitations of previous studies, prospective randomized trials are required to assess the activity of different types of MET inhibitors in patients with NSCLC exhibiting MET dysregulation. Future studies should also aim to identify unique biomarkers and accurate diagnostic platforms for MET-targeted therapeutic strategies to avoid disparities in the evaluation of clinical outcomes.

## Author contributions

Conception and design: FL and LX. Acquisition of data: LX and FW. Critical revision of the manuscript for important intellectual content: FL, LX, and FW. Statistical analysis: LX and FW. Obtain funding: FW. All authors contributed to the article and approved the submitted version.

## Funding

This work was supported by Sichuan Science and Technology Program (No.2021YFQ0030); Tibet Science and Technology Program (XZ202201ZY0002G); 1.3.5 project for disciplines of excellence, West China Hospital, Sichuan University (ZYJC18021) and Post-Doctor Research Project, West China Hospital (2021HXBH074).

## Acknowledgments

The authors thank Lichun Zhong (Laboratory of Pulmonary Immunology and Inflammation, Frontiers Science Center for Disease-related Molecular Network, West China Hospital, Sichuan University) for advising on the data collection.

## Conflict of interest

The authors declare that the research was conducted in the absence of any commercial or financial relationships that could be construed as a potential conflict of interest.

## Publisher’s note

All claims expressed in this article are solely those of the authors and do not necessarily represent those of their affiliated organizations, or those of the publisher, the editors and the reviewers. Any product that may be evaluated in this article, or claim that may be made by its manufacturer, is not guaranteed or endorsed by the publisher.
