# Supplementary material for: MET-targeted therapies for the treatment of non-small-cell lung cancer: A systematic review and meta-analysis
Source: Front Oncol. 2022 Oct 27;12:1013299. doi: 10.3389/fonc.2022.1013299 (PMC9646943; doi:10.3389/fonc.2022.1013299)
Supplement: Supplementary file 1 [file Table_1.docx]

Supplementary Material

**Table S1.** Search terms of pubmed

| **#1** | Search ((c-MET alterations OR c-MET aberrations OR MET amplification OR copy number gain OR MET mutations OR MET exon 14 skipping mutation) OR (TKI resistance) AND (c-MET inhibitors OR c-MET targeted therapy OR antibody-based c-MET inhibitors OR c-MET targeted antibodies) OR c-MET inhibitor combination therapy OR c-MET inhibitor treatment regimen)) |
| --- | --- |
| **#2** | Search (((((((((((((((((((((((((((Pulmonary Neoplasms[Title/Abstract]) OR (Neoplasms, Lung[Title/Abstract])) OR (Lung Neoplasm[Title/Abstract])) OR (Neoplasm, Lung[Title/Abstract])) OR (Neoplasms, Pulmonary[Title/Abstract])) OR (Neoplasm, Pulmonary[Title/Abstract])) OR (Pulmonary Neoplasm[Title/Abstract])) OR (Lung Cancer[Title/Abstract])) OR (Cancer, Lung[Title/Abstract])) OR (Cancers, Lung[Title/Abstract])) OR (Carcinoma, Non Small Cell Lung[Title/Abstract])) OR (Carcinomas, Non-Small-Cell Lung[Title/Abstract])) OR (Lung Carcinoma, Non-Small-Cell[Title/Abstract])) OR (Lung Carcinomas, Non-Small-Cell[Title/Abstract])) OR (Lung Carcinomas, Non-Small-Cell[Title/Abstract])) OR (Non-Small-Cell Lung Carcinoma[Title/Abstract])) OR (Non Small Cell Lung Carcinoma[Title/Abstract])) OR (Non Small Cell Lung Carcinoma[Title/Abstract])) OR (Non Small Cell Lung Carcinoma[Title/Abstract])) OR (Non-Small Cell Lung Cancer[Title/Abstract])) OR (Nonsmall Cell Lung Cancer[Title/Abstract])) OR (Lung Cancers[Title/Abstract])) OR (Pulmonary Cancer[Title/Abstract])) OR (Cancer, Pulmonary[Title/Abstract])) OR (Cancer, Pulmonary[Title/Abstract])) OR (Pulmonary Cancers[Title/Abstract])) OR (Pulmonary Cancers[Title/Abstract])) OR (Cancer of Lung[Title/Abstract]) |
| Final search term: #1 AND #2 | |
